# Neonatal Oxytocin Treatment Ameliorates Autistic-Like Behaviors and Oxytocin Deficiency in Valproic Acid-Induced Rat Model of Autism

**DOI:** 10.3389/fncel.2018.00355

**Published:** 2018-10-09

**Authors:** Yu-Chuan Dai, Hong-Feng Zhang, Michael Schön, Tobias M. Böckers, Song-Ping Han, Ji-Sheng Han, Rong Zhang

**Affiliations:** ^1^Neuroscience Research Institute, Peking University, Beijing, China; ^2^Key Laboratory for Neuroscience, Ministry of Education, National Health and Family Planning Commission, Peking University, Beijing, China; ^3^Department of Neurobiology, School of Basic Medical Sciences, Peking University Health Science Center, Beijing, China; ^4^Fujian Provincial Key Laboratory of Neurodegenerative Disease and Aging Research, Institute of Neuroscience, College of Medicine, Collaborative Innovation Center for Brain Science, Xiamen University, Xiamen, China; ^5^Institute for Anatomy and Cell Biology, Ulm University, Ulm, Germany; ^6^International Graduate School in Molecular Medicine Ulm, Ulm University, Ulm, Germany; ^7^Department of Neurology, Ulm University, Ulm, Germany; ^8^Wuxi HANS Health Medical Technology Co., Ltd., Wuxi, China

**Keywords:** autism spectrum disorder, social behavior defect, repetitive behavior, exogenous supplement, oxytocin therapy

## Abstract

Autism spectrum disorder (ASD) is characterized by impaired social communication and repetitive/stereotyped behaviors. The neuropeptide oxytocin (OXT) plays a critical role in regulating social behaviors in the central nervous system, as indicated in both human and animal studies. We hypothesized that central OXT deficit is one of causes of etiology of ASD, which may be responsible for the social impairments. To test our hypothesis, central OXT system was examined in valproic acid (VPA)-induced rat model of autism (VPA rat). Our results showed that adolescent VPA rats exhibited a lower level of OXT mRNA and fewer OXT-ir cells in the hypothalamus than control rats. Additionally, OXT concentration in cerebrospinal fluid (CSF) was reduced. The number of OXT-ir cells in the supraoptic nucleus (SON) of neonatal VPA rats was also lower. Autistic-like behaviors were observed in these animals as well. We found that an acute intranasal administration of exogenous OXT restored the social preference of adolescent VPA rats. Additionally, early postnatal OXT treatment had long-term effects ameliorating the social impairments and repetitive behaviors of VPA rats until adolescence. This was accompanied by an increase in OXT-ir cells. Taken together, we demonstrated there was central OXT deficiency in the VPA-induced rat model of autism, and showed evidence that early postnatal OXT treatment had a long-term therapeutic effect on the autistic-like behaviors in VPA rats.

## Introduction

Autism Spectrum Disorder (ASD) is a neurodevelopmental disorder characterized by impaired social interaction and communication, as well as repetitive and stereotyped behavior (Bhat et al., 2014). Although genetic factors are strongly indicated to be involved in the onset of ASD (Sebat et al., 2007; Klei et al., 2012; De Rubeis et al., 2014), they are not responsible for all cases. Specific environmental factors, such as prenatally viral infection (Ashwood and Van de Water, 2004; Brown, 2012) or exposure to valproic acid (VPA) (Bromley et al., 2013; Christensen et al., 2013), may increase the risk of ASD in offspring. The etiology of ASD is therefore believed to be a result of interactions between genetic and environmental factors (Hallmayer et al., 2011; Chaste and Leboyer, 2012; Tordjman et al., 2014). Due to high clinical and genetic heterogeneity (Bhat et al., 2014; Ivanov et al., 2015), the pathogenesis of ASD remains unclear, and thus the treatment of ASD is still a challenge. Clinical studies indicate that rehabilitation training can partially alleviate behavioral deficits, but they fail to rescue all of the core symptoms in individuals with ASD (Virués-Ortega, 2010; MacDonald et al., 2014; Tonge et al., 2014). Moreover, specific medication for the treatment of the social impairments or language obstacles in ASD was lacking (McCracken et al., 2002; Bowers et al., 2015). Therefore, a better understanding of the underlying mechanisms of ASD is necessary and which might provide treatment strategies in drug development.

Substantial evidence indicates that the neuropeptide oxytocin (OXT) is a powerful regulator of social behaviors (Stoop, 2012; Caldwell, 2017; Cservenak et al., 2017; Yoshihara et al., 2017). Oxytocin in the brain is synthesized and secreted by the paraventricular nucleus (PVN) and supraoptic nucleus (SON) (Stoop, 2012), and OXT-containing neural projections and OXT receptors (Oxtr) are widely distributed in the brain areas involved in regulating social behavior (Caldwell, 2017; Song and Albers, 2017). *Oxt* (Ferguson et al., 2000; Ferguson et al., 2001) or *Oxtr* (Sala et al., 2011; Pobbe et al., 2012a,b) knockout (KO) mice have been reported to exhibit less social communication and interaction, along with impaired social cognitive memory and other altered behaviors relevant to ASD (Zhang et al., 2017). Central infusion or peripheral injection of OXT has been shown to promote social approach (Lukas et al., 2011; Teng et al., 2016), pair bonding (Bales and Carter, 2003; Bales et al., 2007; Ross et al., 2009), and social memory (Popik and van Ree, 1991; Benelli et al., 1995) in rodents.

Furthermore, reversal of autistic-like behaviors following OXT administration has been observed in monogenic rodent models of autism and patients with ASD. Exogenous OXT administration was shown to rescue social impairments in mice with deletion of *Oxt* (Ferguson et al., 2000), *Oxtr* (Sala et al., 2011), *Cd38* (Jin et al., 2007), *CNTNAP2* (contactin-associated protein-like 2 gene) (Penagarikano et al., 2015), *MAGEL2* (MAGE-like protein 2 gene) (Meziane et al., 2015), or *Oprm1* (mu-type opioid receptor 1 gene) (Gigliucci et al., 2014). In clinical studies, acute OXT administration facilitated social cognition and interaction, ameliorated repetitive behavior, and enhanced language competence in patients with ASD (Hollander et al., 2003; Hollander et al., 2007; Guastella et al., 2010; Hall et al., 2012). However, the effects of repeated dosing of OXT on autistic behaviors remain ambiguous (Kosaka et al., 2012; Tachibana et al., 2013; Dadds et al., 2014; Guastella et al., 2015; Watanabe et al., 2015). Recent studies have revealed that OXT improved social cognition and social preference by enhancing social information processing (Oettl et al., 2016; Raam et al., 2017) and promoting social reward (Hung et al., 2017; Smith et al., 2017).

Although the underlying mechanisms of ASD are still largely unknown, deficiency in the OXT systems has been suggested to be involved in its development (Preti et al., 2014; Zhang et al., 2017) because of the critical role of OXT in regulating social behaviors. Deficits in OXT-immunoreactive (OXT-ir) neurons and peptide levels were observed in several monogenic mouse models of autism, including BTBR mice (Silverman et al., 2010b), *CNTNAP2* (Penagarikano et al., 2015) KO mice and *MAGEL2* (Meziane et al., 2015) KO mice. However, whether central OXT deficiency occurs in autistic models induced by environmental factors is unknown, as is whether OXT administration is capable of ameliorating autistic behaviors in these types of ASD models.

Valproic acid (VPA) is widely used as an antiepileptic drug in clinics (Löscher, 2002). Embryonic exposure of VPA has been reported to be associated with congenital malformations (Ornoy, 2009), cognitive impairments (Meador et al., 2013) as well as ASD (Bromley et al., 2008; Bromley et al., 2013; Smith and Brown, 2014). In animal studies, a single intraperitoneal injection of VPA during the mid-pregnancy of rats was used to produce a rat model of autism in offspring (Schneider and Przewlocki, 2005). Offspring in these cases have been reported to exhibit a series of autistic behaviors, including reduced ultrasonic communication, impaired social interaction, and repetitive/stereotyped behaviors (Roullet et al., 2013; Nicolini and Fahnestock, 2018). Importantly, this autistic model has been widely used to investigate the intrinsic link between some environmental risk factors and the pathogenesis of ASD (Stefanik et al., 2015; Bertelsen et al., 2017; Hara et al., 2017; Wu et al., 2017; Yamaguchi et al., 2017).

In the present study, the VPA-induced rat model of autism was used to investigate the relationship between its behavioral phenotype and the content of central OXT system in the brain. Changes in autistic-like behaviors in VPA rats were examined following exogenous OXT administration.

## Materials and Methods

### VPA-Induced Rat Model of Autism

Male and female Wistar rats weighing 270–290 g were obtained from the Department of Experimental Animal Sciences, Peking University Health Science Center. Animals were housed individually under a regulated environment (23 ± 2°C; 50% ± 10% humidity) with a 12–12 h light-dark cycle. This study was carried out following USA National Institutes of Health Guide for the Care and Use of Laboratory Animals. The protocol was approved by Peking University Animal Care and Use Committee (ethics approval ID, LA2015204).

As previously described, female and male rats were allowed to mate overnight. The day was considered embryonic day 0.5 (E0.5) in the presence of a vaginal plug. The pregnant rats were randomly distributed into two groups: VPA group and control (Ctrl) group. In VPA group, pregnant rats were intraperitoneally injected with VPA (Sigma:P4543, diluted with normal saline to a concentration of 200 mg/ml) at a dose of 600 mg/kg body weight on E12.5 (Schneider and Przewlocki, 2005). The pregnant rats in the control group received the same volume of normal saline at E12.5. After weaning at postnatal day 21 (PND21), offspring of same sex were housed separately with 4–5 per cage.

**FIGURE 1:**
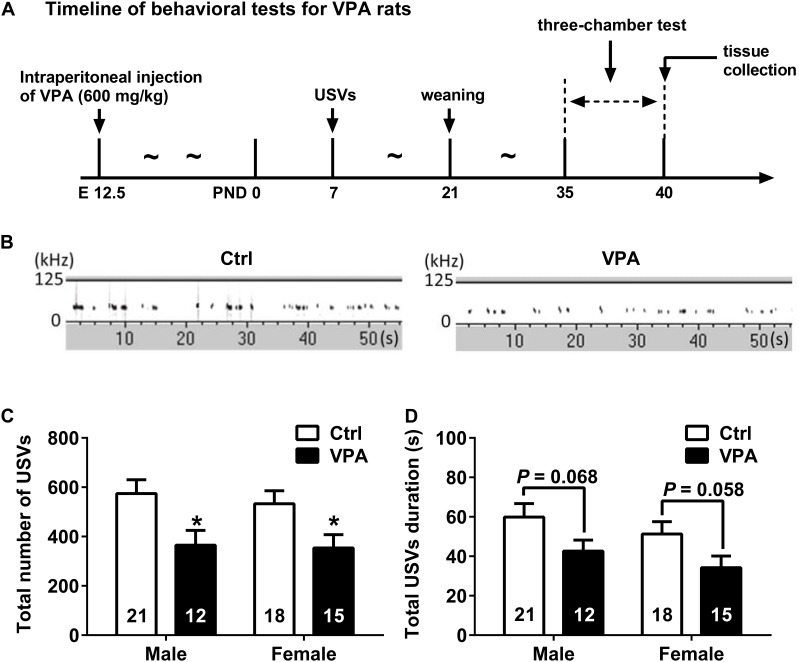
Timeline of behavioral tests and USVs analysis of control and VPA rats. **(A)** Experimental design for behavioral tests. **(B)** Representative spectrograms of USVs recorded from control and VPA pups. **(C)** Both male (*n* = 12) and female (*n* = 15) rats in VPA group emitted fewer USVs in 5 min compared with control group (male *n* = 21, female *n* = 18). **(D)** Total USVs duration tended to be shorter in the VPA group of both sexes. Data are expressed as mean ± SEM, ^∗^*P* < 0.05. Ctrl, control; USVs, ultrasonic vocalizations.

Ultimately, there were three offspring cohorts used in this study. The first cohort was used for assessment of social behaviors, anxiety, self-grooming behavior, and the immunohistochemical and biochemical analysis of central OXT system for each group (see **Figure 1A** for experimental procedure). Analyses were conducted on both male and female offspring in this cohort. The second cohort was used to evaluate the effect that a single intranasal OXT administration had on rat social behaviors of VPA rats at PND40. The third cohort was used to evaluate the effect of the postnatal OXT treatment (7 daily subcutaneous injections beginning from PND0) on autistic-like behaviors of VPA rats. We also analyzed the number of OXT-ir cells in the hypothalamus of VPA rats following the treatment (see **Figure 6A** for experimental procedure). Only male offspring were used in second and third cohorts because VPA rats had no gender differences in phenotypes (see “VPA Rats Displayed Social Impairments” and “VPA Rats Exhibited Central OXT System Deficiency”)

### Isolation-Induced Ultrasonic Vocalizations (USVs)

Changes in social communication of pups were tested by analyzing isolation-induced USVs as reported previously. USVs were recorded on PND7 between 18:00 and 22:00 in a quite environment with dim light. Briefly, pups were individually removed from the home cage and gently transferred to the test cage on a heating pad (37°C). USVs were recorded for 300 s for each pup and collected by an ultrasonic microphone (CM16/CMPA, Avisoft Bioacoustics, Berlin, Germany) that hung 25 cm above the cage floor. The connected amplifier (AUSG-116H, Avisoft Bioacoustics, Berlin, Germany) was set at a sampling frequency of 250 kHz with a 125 kHz low-pass filter. The recorded files were analyzed with Avisoft SASLab Pro (Version 4.52) using fast Fourier transform (512 FFT-length, 100% frame size, Hamming window, 50% time-window overlap) (Xu et al., 2015).

### Behavioral Tests

#### Sociability Test

**FIGURE 2:**
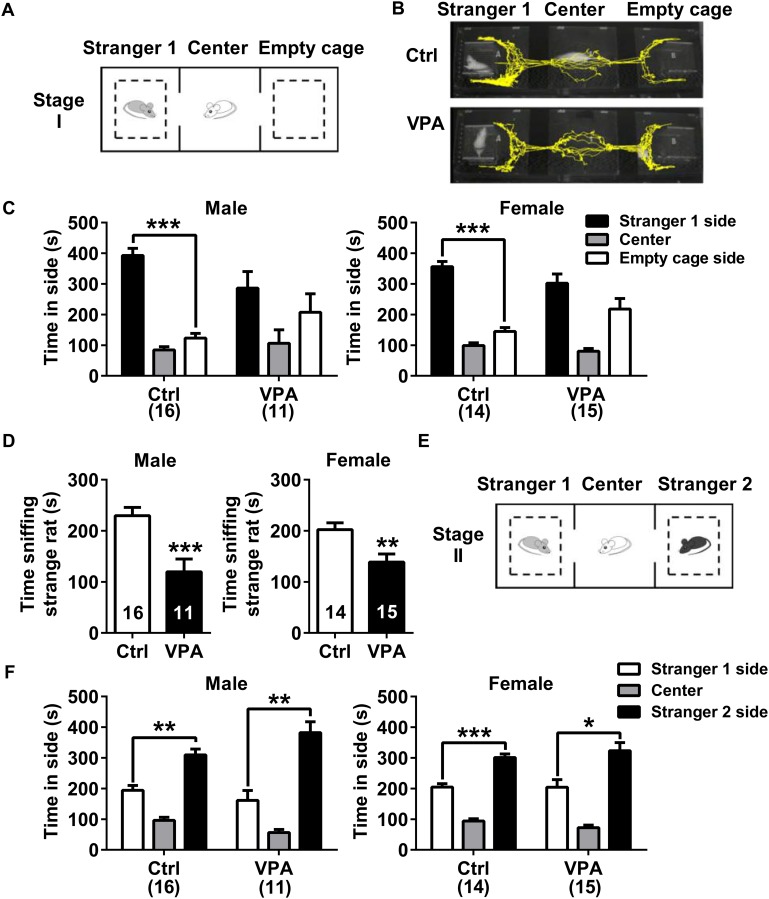
Social interaction in control and VPA rats. **(A–D)** Stage I of three-chamber sociability test (social preference). **(A)** The paradigm for testing social preference. **(B)** Representative traces from control and VPA rats exploring the two sides (Stranger 1 and empty cage). Both the males (*n* = 11) and females (*n* = 15) in the VPA group exhibited deficits in social preference **(C)** and spent less time sniffing the stranger **(D)** than the control group (male *n* = 16, female *n* = 14). **(E**,**F)** Stage II of three-chamber sociability test (social novelty). **(E)** The paradigm for testing social novelty. **(F)** No differences were observed between VPA and control rats of both sexes in the social novelty test. Data are expressed as mean ± SEM, ^∗^*P <* 0.05, ^∗∗^*P <* 0.01, ^∗∗∗^*P <* 0.001. Ctrl, control.

Social preference and social novelty of adolescent rats were evaluated by the three-chamber sociability test on PND 35-40, as documented in past study (Zhang et al., 2015). The three-chamber apparatus comprises three rectangular plexiglass chambers (40 cm × 34 cm × 24 cm) with each side chambers connected to the central chamber by a corridor. The assay began with a 5 min habituation during which the subject rat was placed in the central chamber and allowed to freely explore the empty apparatus. Two successive stages (Stage I and Stage II) were followed. Stage I for social preference: an unfamiliar, weight and sex matched Wistar rat (Stranger 1) was locked in a wire cage and placed in one of the side chambers. An identical empty cage was placed in the other side chamber. Then the subject rat was placed in the central chamber and allowed to explore the side chambers for 10 min (paradigm showed in **Figure 2A**). Stage II for social novelty: another unfamiliar, weight and sex matched Wistar rat (Stranger 2) was placed in the empty cage used in the Stage I. The subject rat was then allowed to access to both sides freely for 10 min (paradigm showed in **Figure 2E**). During the experiment, the time spent in each of the three chambers was automatically recorded. Movement paths were video recorded and track plots were analyzed with an image video-tracking system (Smart2.5, PanLab, Harvard Apparatus, United States). To minimize the impact from residual rat odors, the entire apparatus was thoroughly cleaned with 70% ethanol at the beginning of each trial. All behavior studies were carried out during the dark cycle.

#### Light/Dark Box Test

The light/dark box comprised an illuminated (40 cm × 34 cm × 24 cm) and a dark (40 cm × 34 cm × 24 cm) compartment separated by a partition with a door. The test was carried out to assess the anxiety level of adolescent rats. At the beginning of the experiment, the subject was placed into the light side and allowed to explore the apparatus for 5 min. Time spent in the light side was automatically recorded. At the beginning of each trial, the entire apparatus was thoroughly cleaned with 70% ethanol.

#### Self-Grooming Behavior

The self-grooming test is a paradigm that measures the level of stereotyped behavior of rodents (Kalueff et al., 2016). During habituation, animals were placed into an empty cage similar to the home cage and encouraged to explore for 10 min. Rat behavior was then video-taped for 10 min and the total self-grooming time was calculated. In the present study, self-grooming behaviors include: (1) wiping the nose, face, head, and ears with forepaws; (2) licking body, anogenital area, and tail (Kalueff et al., 2016).

### OXT Treatment

#### Single Intranasal Treatment

To verify the pro-social effect of OXT treatment in adolescent VPA rats, a single dose of OXT (ProSpec-Tany TechnoGene Ltd, Rehovot, Israel) or normal saline was administered intranasally in control and VPA rats 30 min before the social preference test. OXT was dissolved in normal saline to a concentration of 1 μg/μl. Gently and fleetingly, 20 μl of saline or OXT solution was applied bilaterally on the rhinarium with a P10 pipette. The rat was held with its nose up for 1 min to ensure that liquid was inhaled and diffused into nostrils thoroughly (Lukas and Neumann, 2012; Neumann et al., 2013). Rats were then released to their home cage until the test started.

#### Repeated Subcutaneous Dosing at an Early Postnatal Stage

To achieve long-term therapeutic effect on social behaviors, we attempted to administer OXT at an early postnatal stage since it had been suggested to have long-term effects on social behaviors in other rodent models of autism. Within 24 h of birth, VPA pups received randomly either a single subcutaneous injection of OXT (3 μg/20 μl in normal saline) or 20 μl normal saline. Subsequently, an identical dose of OXT solution or normal saline was administered daily in the following 6 days (Meziane et al., 2015). Control pups only received equal volume of normal saline during the first week after birth. Isolation-induced USVs of rat pups was evaluated on PND7. After weaning, offspring from same litter were housed together (3–4 pups per cage) and were subjected to the rest of experiments from PDN35 to PND40 (see **Figure 6A** for experiment procedure).

#### Tissue Collection

For determining OXT mRNA and peptide levels, rats were anesthetized by intraperitoneal injection with 10% chloral hydrate (300 mg/kg body weight) before sampling.

#### Cerebrospinal Fluid (CSF) Collection

Under general anesthesia, rat heads were immobilized in a stereotaxic frame (David Kopf Instruments, CA, United States) in the flat-skull position. After incision of the skin on the back of neck and blunt dissection of dorsal muscles, the Cerebrospinal **F**luid (CSF) (∼150 μl) was collected from the cisterna magna. All CSF samples were stored at –80°C until assayed. According to our preliminary experiments, 450 μl of CSF was required to assess OXT levels using an oxytocin ELISA kit (see below). Therefore, every three CSF samples of the same group and of the same sex were pooled and analyzed as a single sample. Because the number of CSF samples was limited, the data from both sex were analyzed together.

#### Blood-Sample Collection

Following decapitation, trunk blood (∼5 ml) was collected rapidly into a clean tube containing Aprotinin (Sigma-Aldrich, United States) at a final concentration of 500 KIU/ml of blood. The blood samples were maintained at room temperature for 30 min, and then centrifuged at 1600 g for 15 min at 4°C. The serum was separated and divided into aliquots of 650 μl and stored at –80°C.

#### Brain-Tissue Collection

Rats were decapitated and the brain was quickly removed and frozen in liquid nitrogen for 20 s. The frozen brains were stored at -80°C until the PVN and SON were dissected from the hypothalamus. According to the Paxinos and Watson Rat Brain Atlas, bilateral punches of the PVN and SON (1 mm and 1.5 mm in diameter, respectively) were taken at –20°C and then store at –80°C until assayed.

#### Determination of OXT mRNA Levels

Total mRNA in unilateral punches of PVN or SON was extracted with TRIzol reagent (Invitrogen, Carlsbad, CA, United States) according to the manufacturer’s instructions and digested with DNase (Promega, Madison, WI, United States) to remove DNA contamination. For cDNA synthesis, reverse-transcription of the mRNA was performed with the PrimeScript RT-PCR kit (TaKaRa, Dalian, China). Gene expression analysis of OXT and the house-keeping gene (β-actin) was evaluated using TaqMan Gene Expression Assays (assay ID: OXT, Rn00564446_g1; β-actin, Rn00667869_m1) following standard qPCR procedure: 2 min at 50°C, 10 min at 95°C, 40 cycles of 15 s at 95°C and 1 min at 60°C. The results were calculated using the 2^-ΔΔCT^ method with β-actin as the endogenous reference gene, and ultimately converted to fold changes versus control.

#### Detection of OXT Levels in the CSF and Serum

OXT content in the CSF and serum was assayed using a commercially available ELISA kit (Enzo Life Sciences, PA, United States). All samples were subjected to prior extraction with acetone and petroleum ether according to the product manual. The ELISA kit was highly sensitive (detecting limit: 15.0 pg/ml for OXT) with little cross-reactivity (less than 7.5%) with arg^8^-vasopressin. The intra-assay CV for OXT was 10.2%.

#### Immunohistochemistry

Rats were anesthetized by intraperitoneal injection with 10% chloral hydrate (300 mg/kg body weight). They were then transcardially perfused with normal saline (37°C) and 4% paraformaldehyde in 0.1 M phosphate buffer (PFA, 4°C, pH 7.4). Brains were quickly removed and post-fixed in 4% PFA at 4°C for 12 h. Brains were sequentially soaked in 20 and 30% sucrose in 0.1 M phosphate buffer until saturated for cryoprotection. Each brain was coronally sectioned into 30-μm thick slices (20 μm for rat pups) using a freezing microtome and sections were then stored at -20°C in anti-freezing solution. OXT staining was performed by the modified ABC (avidin-biotin-peroxidase complex) immunohistochemical technique. Before each stage of the process, sections were rinsed in 0.01 M phosphate buffered saline (PBS, pH 7.2) three times (5 min each). The procedures were performed as follows: brain sections were fixed in 4% PFA for 10 min and then, pre-incubated in 0.3% hydrogen dioxide for 30 min at 37°C to eliminate endogenous peroxidase activity. Thereafter, the sections were incubated with 10% normal goat serum and 0.3% Triton X-100 in 0.01 M PBS for 30 min at 37°C, followed by incubation with rabbit anti-OXT antibody (Abcam, United States., 1:2000 dilution in 0.01 M PBS containing 1% normal goat serum and 0.3% TritonX-100) at 4°C for 48 h. The sections were then incubated in the anti-rabbit secondary immunoglobulin G (IgG) conjugated to horseradish peroxidase (OriGene Technologies, Inc., Beijing, China) at 37°C for 30 min. Immunoreactivity was visualized using the 3,3’-diaminobenzidine kit (OriGene Technologies, Inc., Beijing, China) to produce a brown nuclear reaction product. Lastly, the free-floating sections were mounted onto gelatin coated glass slides, dehydrated in a series of alcohols, cleared in xylene, and placed under a coverslip with gum.

Six sections containing the PVN or SON (between -0.8 and -2.1 mm from the Bregma) were selected every 150 μm for OXT immunohistochemical analysis. The total number of OXT-ir cells in all six sections was counted as the data for each sample. OXT-ir cells were photographed bilaterally in the PVN and SON under a Leica automated DMI-inverted microscope (Leica DMI 4000B). Coronal sections were matched between samples.

#### Statistics

The number of animal used in each experiment is mentioned in figure legends. Statistical analyses were performed and graphs were generated by GraphPad Prism 7 (GraphPad Software Inc., San Diego, CA, United States). All results are expressed as mean ± SEM. Unpaired Student’s *t* test or Mann-Whitney U test was used for behavioral and biochemical comparisons between groups (VPA versus control) or treatment (normal saline versus OXT). The three-chamber social test was analyzed with paired *t* test to determine within-group side preference. Two-way ANOVA followed by a Tukey *post hoc* test was used to correct for multiple comparisons. Statistical significance was set at *P* < 0.05 (two-tailed) in all tests.

## Results

### VPA Rats Displayed Social Impairments

Social deficits of VPA rats were assessed by USVs and three-chamber sociability test (see **Figure 1A** for experiment procedure). Results of isolation-induced USVs test (representative USVs spectrograms showed in **Figure 1B**) showed that the VPA group emitted fewer USVs than the control group (unpaired *t* test; male *P* < 0.05, female *P* < 0.05, **Figure 1C**). Additionally, we observed a trend toward differences in total USVs duration between the VPA pups and control pups (unpaired *t* test; male *P* = 0.068, female *P* = 0.058, **Figure 1D**). These data indicated a social communication impairment in infant of VPA rats.

In three-chamber sociability test (social preference, see illustration in **Figure 2A** and representative movement traces in **Figure 2B**), the control rats spent more time in the side with Stranger 1 than in the empty cage (paired *t* test; male *P* < 0.001, female *P* < 0.001, **Figure 2C**). In contrast, VPA rats seemed to lose interest in social interaction with conspecifics, spending a comparable time exploring the cage containing Stranger 1 rat and the empty cage (paired *t* test; male *P* = 0.4699, female *P* = 0.2082, **Figure 2C**). Furthermore, the social sniffing time was significantly lower in VPA rats than in controls (unpaired *t* test; male *P* < 0.001, female *P* < 0.01, **Figure 2D**). These data indicated that social interaction was impaired in adolescence of VPA rats. In contrast, both VPA male and female rats exhibited normal social novelty (see illustration in **Figure 2E**), as they spent more time in proximity to the Stranger 2 rat than the Stranger 1 rat (paired *t* test; male *P* < 0.01, female *P* < 0.05, **Figure 2F**).

### VPA Rats Exhibited Central OXT System Deficiency

**FIGURE 3:**
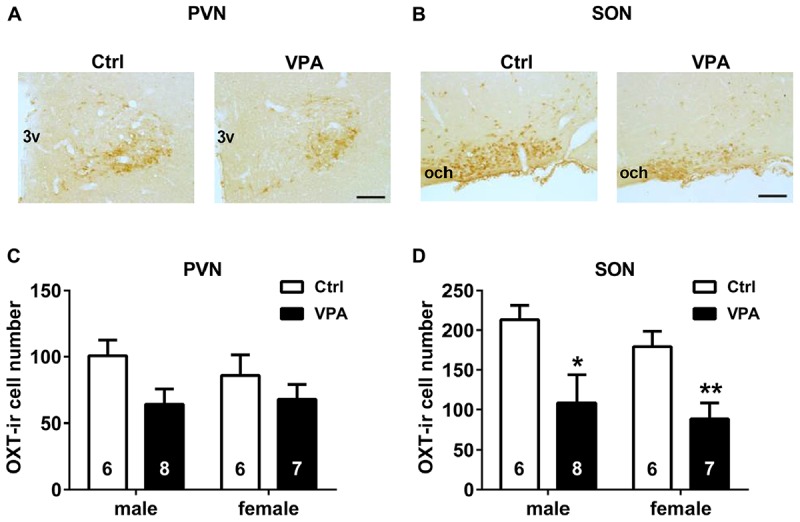
Neonatal VPA rats had fewer OXT-ir cells in the hypothalamus than controls. Representative images (bar = 100 μm) of OXT staining in the PVN **(A)** and SON **(B)** of both control and VPA rats. In the PVN **(C)**, the number of OXT-ir cells did not differ between groups. But in the SON **(D)**, they were less in the VPA rats than controls. Data are expressed as mean ± SEM (control: male *n* = 6, female *n* = 6; VPA: male *n* = 8, female *n* = 7), ^∗^*P <* 0.05, ^∗∗^*P <* 0.01. Ctrl, control; PVN, paraventricular nucleus; SON, supraoptic nucleus; 3v, third ventricle; and och, optic chiasm.

In infancy, the number of OXT-ir cells in SON was lower in VPA pups than in control pups for both males and females (representative images of OXT staining showed in **Figures 3A,B**; unpaired *t* test; male *P* < 0.05, female *P* < 0.01, **Figure 3D**). In the PVN, the number of OXT-ir cells in male VPA pups was fewer than in male controls. In contrast, the females did not display any significant differences (male *P* = 0.05, female *P* = 0.3560, **Figure 3C**).

**FIGURE 4:**
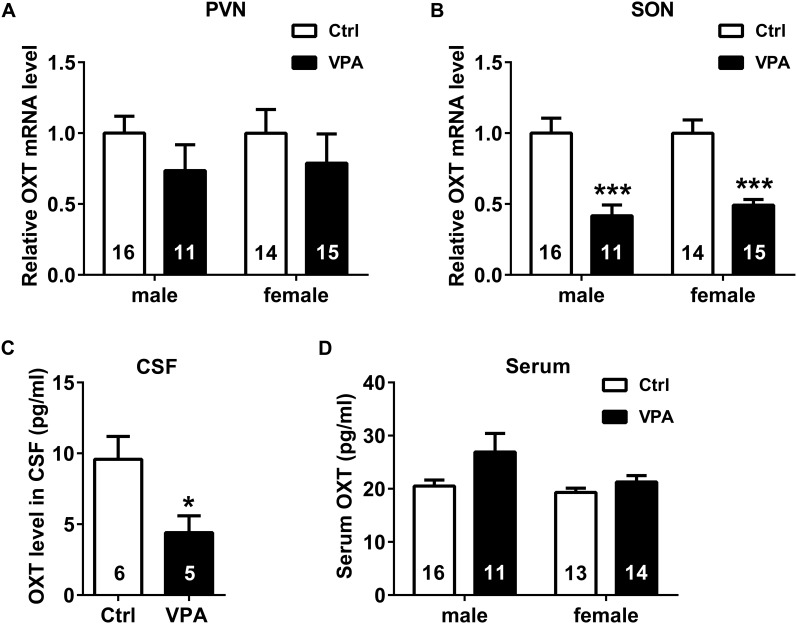
Adolescent VPA rats exhibited deficient central OXT mRNA and peptide levels. **(A,B)** Compared to control rats (male *n* = 16, female *n* = 14), lower OXT mRNA levels were observed in the SON, but not in the PVN of male (*n* = 11) and female (*n* = 15) VPA rats. **(C)** OXT levels in the CSF were also lower in VPA rats (control *n* = 6, VPA *n* = 5). **(D)** Serum OXT levels did not differ significantly between control (male *n* = 16, female *n* = 13) and VPA (male *n* = 11, female *n* = 14) rats. Data are expressed as mean ± SEM, ^∗^*P <* 0.05, ^∗∗∗^*P <* 0.001. Ctrl, control; PVN, paraventricular nucleus; SON, supraoptic nucleus; and CSF, cerebrospinal fluid.

OXT mRNA levels were significantly lower in the SON of male and female VPA rats (unpaired *t* test; male *P* < 0.001, female *P* < 0.001, **Figure 4B**), but not in the PVN (male *P* = 0.2153, female *P* = 0.4350, **Figure 4A**). OXT levels in the CSF of VPA rats were also lower than in control rats (unpaired *t* test, *P* < 0.05, **Figure 4C**). In contrast, serum OXT levels showed no significant differences between VPA and control rats in either sex (unpaired *t* test; male *P* = 0.0542, female *P* = 0.1968, **Figure 4D**). In summary, by using immunohistochemical, RT-PCR and ELISA analyses, we have shown central OXT deficiency in VPA rats.

### Effect of Acute OXT Treatment on Social Behavior

**FIGURE 5:**
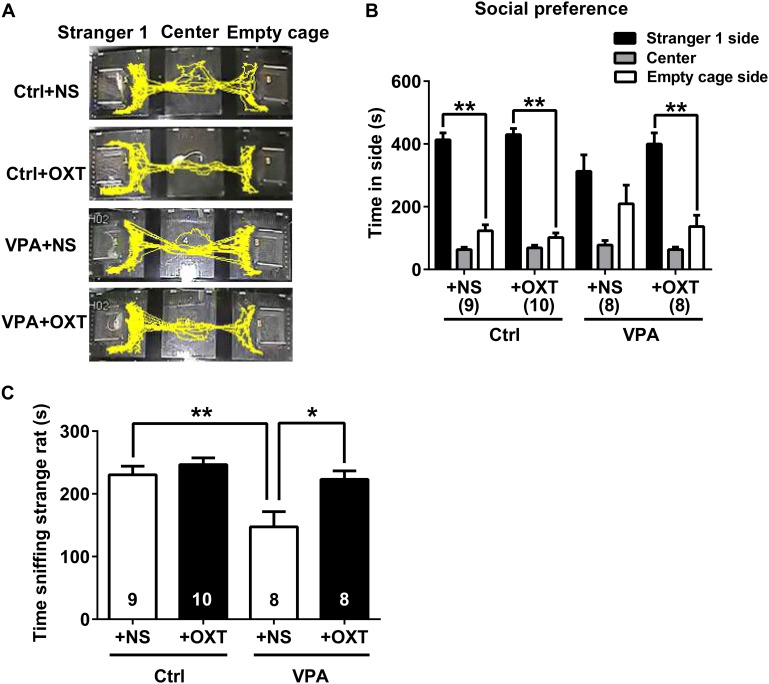
Acute OXT administration ameliorated social deficits of VPA rats. **(A)** Representative traces from the three-chamber social preference test for control and VPA rats treated with normal saline (NS) or OXT. **(B,C)** A single dose of intranasal OXT treatment on VPA rats improved their social preference **(B)** and prolonged the time spent sniffing conspecifics **(C)**. Data are expressed as mean ± SEM (Ctrl + NS *n* = 9, Ctrl + OXT *n* = 10, VPA + NS *n* = 8, VPA + NS *n* = 8), ^∗^*P <* 0.05, ^∗∗^*P <* 0.01. Ctrl, control.

Sociability of adolescent VPA rats was assessed using the three chamber sociability test 30 min after the intranasal OXT treatment. The results showed that impaired sociability in VPA rats was partially reversed following the OXT treatment, characterized by spending more time in the side with Stranger 1 than with the empty cage (see representative movement traces in **Figure 5A**; paired *t* test for Stranger 1 vs. empty cage, VPA + OXT *P* < 0.01, **Figure 5B**), and by spending more time in social sniffing (VPA + OXT vs. VPA + NS, *P* < 0.05, **Figure 5C**).

### Early Postnatal OXT Treatment Exhibited Long-Term Behavioral Effect

**FIGURE 6:**
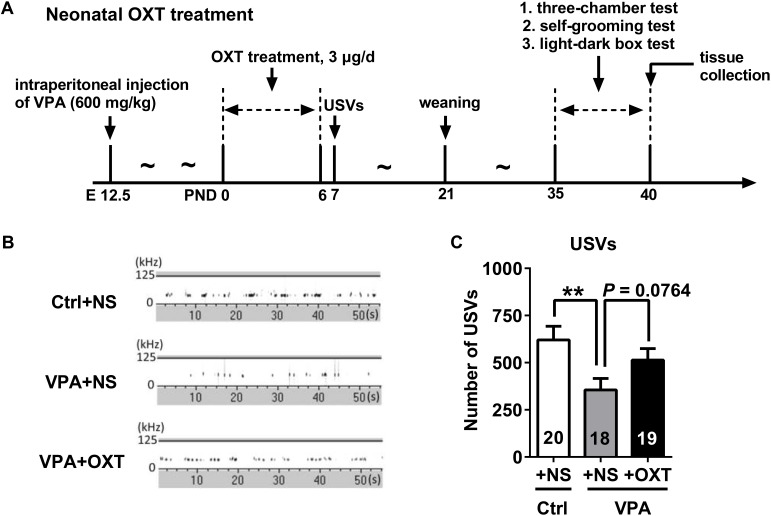
Early postnatal OXT administration had a tendency to improve the number of USVs emitted by VPA pups. **(A)** Experimental design for measuring the effect of neonatal OXT treatment. **(B,C)** Representative USV spectrogram recorded from rat pups **(B)** and total number of USVs **(C)**. Data are expressed as mean ± SEM (Ctrl + NS *n* = 20, VPA + NS *n* = 18, VPA + OXT *n* = 19), ^∗∗^*P <* 0.01. Ctrl: control; USVs, ultrasonic vocalizations.

For testing the therapeutic effect of early OXT treatment on the VPA rats, postnatal OXT supplement was administrated to neonate VPA rats and then sociability, self-grooming and anxiety were evaluated during adolescence (see **Figure 6A** for experiment procedure).

**FIGURE 7:**
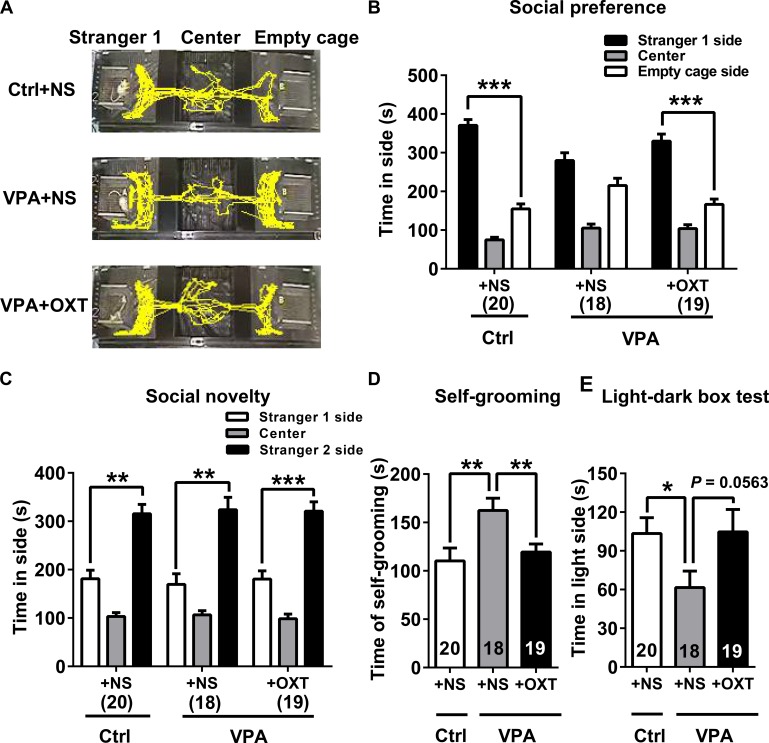
Early postnatal OXT administration had a long-term therapeutic effect on autistic behaviors of VPA rats. **(A,B)** Social preference of VPA rats was normal following early postnatal OXT treatment. **(C)** Social novelty of VPA rats was not affected. **(D)** Time spent in self-grooming by VPA rats was lowered following early postnatal OXT administration. **(E)** The time in the light side of light-dark box was not significantly different following OXT treatment during the light-dark box test. Data are expressed as mean ± SEM (Ctrl + NS *n* = 20, VPA + NS *n* = 18, VPA + OXT *n* = 19), ^∗^*P <* 0.05, ^∗∗^*P <* 0.01, ^∗∗∗^*P <* 0.001. Ctrl, control.

Analysis showed that early postnatal OXT treatment had a tendency to improve the total number of USVs for VPA pups (representative USVs spectrograms showed in **Figure 6B**; unpaired *t* test; Ctrl + NS vs. VPA + NS *P* < 0.01, VPA + OXT vs. VPA + NS *P* = 0.0764, **Figure 6C**). Additionally, we observed that chronic OXT treatment in the early stage of life rescued in certain degree some of the social deficits found in adolescent VPA rats in three-chamber sociability test (see representative movement traces in **Figure 7A**). Adolescent rats in the VPA + OXT group spent more time exploring around the Strange 1 than the empty cage (*P* < 0.001, **Figure 7B**), indicating a normal social preference. For social novelty, rats in VPA + OXT group spent more time near the Strange 2 than the Stranger 1, suggesting that early postnatal OXT treatment had no influence on social novelty in VPA rats (**Figure 7C**).

In the present study, the duration of self-grooming was significantly longer in VPA rats than in controls (unpaired *t* test for VPA + NS vs. Ctrl + NS, *P* < 0.01, **Figure 7D**). The light-dark box test (**Figure 7E**) revealed that VPA rats spent significantly less time in the light side than control rats did (unpaired *t* test VPA + NS vs. Ctrl + NS, *P* < 0.05), indicating a higher level of anxiety in VPA rats. Intriguingly, early postnatal intervention of OXT significantly reduced the time that adolescent VPA rats spent in self-grooming (unpaired *t* test for VPA + OXT vs. VPA + NS, *P* < 0.01, **Figure 7D**). Further, their anxiety levels also tended to be lower following OXT treatment, although with marginal significance (unpaired *t* test for VPA + OXT vs. VPA + NS, *P* = 0.0563, **Figure 7E**).

### Effect of Early Postnatal OXT Treatment on OXT-ir Cell Numbers

**FIGURE 8:**
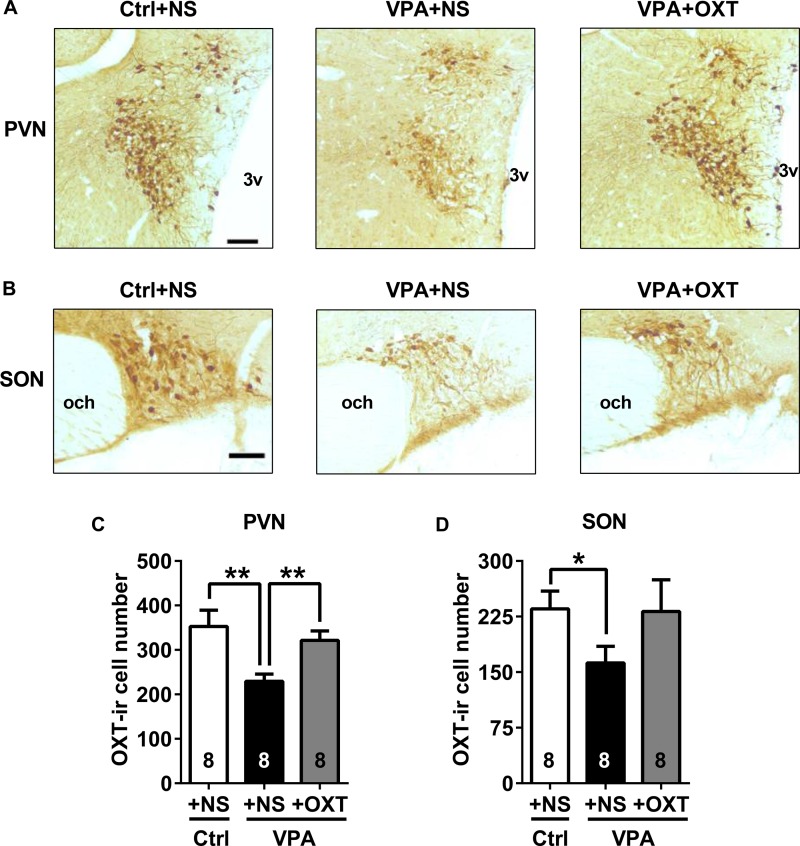
Effects of early postnatal OXT administration on the number of OXT-ir cells in adolescent rats. **(A**,**B)** Representative images (bar = 100 μm) of OXT staining in the PVN **(A)** and SON **(B)** of control and VPA rats. **(C**,**D)** Fewer OXT-ir cells were observed in the PVN **(C)** and SON **(D)** of adolescent VPA rats than controls. Postnatal OXT treatment restored the number of OXT-ir cells in the PVN **(C)**, but not in the SON **(D)**. Data are expressed as mean ± SEM (Ctrl + NS *n* = 8, VPA + NS *n* = 8, VPA + OXT *n* = 8). ^∗^*P <* 0.05, ^∗∗^*P <* 0.01. Ctrl, control; PVN, paraventricular nucleus; SON, supraoptic nucleus; 3v, third ventricle; and och, optic chiasm.

In adolescence, the number of OXT-ir cells in both the PVN and SON of VPA rats were significantly less than in control rats (representative images of OXT staining showed in **Figures 8A,B**; unpaired *t* test for VPA + NS vs. Ctrl + NS, PVN *P* < 0.01, SON *P* < 0.05, **Figures 8C,D** respectively). Analysis showed that the numbers of OXT-ir cells were significantly restored in the PVN of VPA rats following postnatal OXT treatment (unpaired *t* test for VPA + OXT vs. VPA + NS, *P* < 0.01, **Figure 8C**). A similar trend was seen in the SON, however, the increase in OXT-ir cells following OXT treatment did not reach statistical significance (unpaired *t* test for VPA + OXT vs. VPA + NS, *P* = 0.1740, **Figure 8D**).

## Discussion

Because of the close relationship between OXT and social behavior, we examined the role of OXT in the VPA-induced rat model of autism. Through a series of behavioral tests, we confirmed autistic-like behaviors in VPA rats. Here, we report for the first time that central OXT system is impaired in the VPA rats. Compared to controls, mRNA level and peptide concentration in both male and female VPA rats were lower in the hypothalamus. The peptide level was also decreased in CSF. OXT-ir cells were also deficient in the hypothalamus of VPA treated neonates and adolescents. Additionally, acute intranasal OXT administration improved the impaired social behavior of adolescent VPA rats. Another finding is that early neonatal OXT manipulation had a long-term therapeutic effect on autistic-like behaviors in VPA rats, accompanied by restoration of OXT-ir cells in the hypothalamus. Our results hinted that OXT plays a crucial role in the pathogenesis of social deficits in VPA rats.

ASD is a typical male-biased neurodevelopmental disorder, with a male-to-female ratio of approximately 4 to 1 (Constantino and Charman, 2012; VanderLaan et al., 2015). In contrast, our study showed that both male and female VPA rats displayed similar degree of social deficits, indicating no sex bias for this rodent autistic model. This is in accordance with clinical epidemiological findings that the sex ratio of childhood autism was 1.2 (boys to girls) for children exposed to VPA *in utero* (Rasalam et al., 2005; Christensen et al., 2013). These findings indicated that prenatal exposure to VPA may affect the male and female offspring similarly.

The three-chamber sociability test indicated that the social preference of VPA rats was impaired, while their social novelty was normal. To a certain extent, the latter behavior reflects the ability of animals for social recognition (Silverman et al., 2010a; van der Kooij and Sandi, 2012). The results indicated that VPA rats had normal social recognition and social memory. Our findings are supported by a previous study in which the VPA exposure at the same dose (600 mg/kg body weight) on E12.5 led to abnormal social preference and social communication, but normal social novelty in mice (Moldrich et al., 2013). This suggests that social interaction and social memory may be two relatively independent social behaviors with different level of tolerability to environmental changes. In addition, one type of behavioral impairment does not affect the other.

Central OXT deficiency in VPA rats might result from pharmacological action of VPA. VPA is a histone deacetylase (HDAC) inhibitor (Kataoka et al., 2013; Moldrich et al., 2013) and can increase acetylation of histone H4 in cultured cells in a dose-dependent manner (Gottlicher et al., 2001) Changes in histone acetylation can affect chromatin structure reorganization and interfere with gene expression. Similarly, *in utero* exposure to VPA resulted in an increased level of acetylated histones in mouse embryo (Tung and Winn, 2010; Kataoka et al., 2013), which is strongly relevant to postnatal autistic-like behaviors (Kataoka et al., 2013; Moldrich et al., 2013). VPA might interrupt the regulation in the upstream of the embryonic *oxt* gene expression during the critical window of developing brain. Additionally, VPA could also disturb Wnt (Wang et al., 2015; Qin et al., 2016) and GABAergic (Löscher, 2002; Olexova et al., 2016) signaling pathways which play important roles in the progress of cell proliferation and differentiation (Jung et al., 2008), as well as the establishment of neural networks (Baltz et al., 2010) in the developing brain. Previous studies have shown that rats prenatally exposed to VPA had fewer neurons in several brain regions (such as prefrontal and somatosensory cortices) during infancy and adolescence (Rodier et al., 1996; Kataoka et al., 2013), which may contribute to the lower brain-weight and thinner cortex of VPA rats (Mychasiuk et al., 2012; Favre et al., 2013). These may partially explain our findings that the number of OXT-ir cells in the hypothalamus of VPA rats were abnormally low.

It should be pointed out that central OXT dysfunction is not limited to the VPA-induced autistic model. In several models of monogenic or multigenic heritable forms of autism, central OXT levels were also reported to be dysregulated. These models included BTBR mice, and *CNTNAP2*, *MAGEL2*, and *Oprm1* knockout (KO) mice. BTBR mice displayed reduced social interaction (Bales et al., 2014), accompanied with higher OXT content in the PVN of the hypothalamus (Silverman et al., 2010b). In contrast, the number of OXT-ir neurons in the PVN, as well as the level of OXT in the whole brain, were decreased in *CNTNAP2* KO mice (Penagarikano et al., 2015). Similarly, lower OXT levels in nucleus accumbens (NAc) were reported in *Oprm1* KO mice (Becker et al., 2014). *MAGEL2* is believed to be involved in the pathogenesis of ASD. Previous studies have shown that OXT content in hypothalamus was significantly lower in newborn mice lacking *MAGEL2* (Schaller et al., 2010). Conversely, in adult *MAGEL2^-/-^* mice, increased number of OXT-ir neurons and peptide levels were observed in the hypothalamus and hypophysis, respectively (Meziane et al., 2015). Although the above-mentioned autism models vary in etiology, they share a certain degree of impaired social behaviors associated with central OXT defects, suggesting dysregulation of OXT pathway may be the common cause of some of the social deficits.

Unexpectedly, our findings of lower OXT mRNA level in VPA rats were inconsistent with the results reported in a previous study, as they observed increased OXT mRNA levels in the PVN and SON of VPA rats (Stefanik et al., 2015). It should be noted that the VPA rats in that study were adults when OXT expression was measured, while the rats used in the present study were still in adolescence. Additionally, their analyses were under sex-mixed manner. Rats might experience a dramatically physiological change when transitioning from adolescence to adulthood. Sex hormones, like estrogen, secreted by adult rats could regulate *oxt* expression in the brain (Dellovade et al., 1999; Nomura et al., 2002; Murakami et al., 2011). These confounding factors may ultimately influence OXT expression.

Similar to the findings of a recent study (Hara et al., 2017), we also observed that a single intranasal OXT could restore the social preference deficits of VPA rats. The pro-social effects of nasal OXT manipulation have been confirmed in animal studies (Bales et al., 2014; Calcagnoli et al., 2015; Simpson et al., 2017) and clinical studies (Hollander et al., 2003; Andari et al., 2010; Watanabe et al., 2015; Yatawara et al., 2016). Although the underlying mechanism is unclear, immunohistochemical staining of c-Fos (Jia et al., 2008; Hara et al., 2017) and imaging studies (Bethlehem et al., 2013; Rilling et al., 2014) have shown that nasal administration of OXT activates social behavior-relevant areas in the brain. However, an acute exogenous OXT supplementation only produced a temporary pro-social effect (Penagarikano et al., 2015; Hara et al., 2017), probably because the half-life of OXT in the brain is relatively short (Jones and Robinson, 1982; Mens et al., 1983).

Thus, we report the first trial for the treatment of VPA rats with OXT at the neonatal stage. Results showed that neonatal OXT manipulation in the first week after birth improved the social preference and decreased stereotyped behavior in the VPA rats. We further found that the number of OXT-ir cells in the PVN was no longer abnormally low. This long-lasting improvement of postnatal OXT intervention on social behavior and OXT-ir neurons has also been observed in prairie voles (Bales and Carter, 2003; Yamamoto et al., 2004), mandarin voles (Jia et al., 2008), mice (Mogi et al., 2014), and monogenic mouse models of autism (Meziane et al., 2015; Penagarikano et al., 2015). Studies have suggested that OXT promotes neuron proliferation and differentiation. Incubation with OXT *in vitro* (0.01 μM, on the 8th day of neural induction) improves the proliferation and neural differentiation of mouse adipose tissue-derived stem cells (ADSCs) (Jafarzadeh et al., 2014). Rodent studies have revealed that intraperitoneal (1 mg/kg body weight) or hippocampal (1 ng) injections of OXT enhances neurogenesis in the adult hippocampus (Leuner et al., 2012; Sanchez-Vidana et al., 2016), and this is associated with activation of oxytocin receptors (Oxtr) expressed in CA3 pyramidal neurons (Lin et al., 2017). OXT binding to Oxtr induces the release of Ca^2+^ from intracellular stores (Gimpl and Fahrenholz, 2001; Noiseux et al., 2012), and the activation of intracellular Ca^2+^ signaling pathway can further promote cell differentiation and maturation (Batut et al., 2005; Leclerc et al., 2012). In rodents, Oxtr synthesis begins during the embryonic stage and mature Oxtr are expressed in the hypothalamus after birth (Yoshimura et al., 1996; Wang and Young, 1997). In the early stage of life, OXT neurons mainly express the Oxtr (without vasopressin receptors) and response to their own peptides (Freund-Mercier et al., 1994; Hurbin et al., 1998; Chevaleyre et al., 2000). Thus, postnatal OXT administration might activate the Oxtr on the OXT neurons, subsequently enhancing their proliferation. From these results, it is likely that amelioration in autistic behaviors was associated with the promotion of OXT neuronal development. Additionally, the c-Fos staining showed that a single intraperitoneal OXT injection given after birth activated the neurons in several brain areas, especially the SON of male prairie voles (Cushing et al., 2003). These data indicate that neonatal OXT played a role in modulating the activity of neurons in the developing brain resulting in remodeling of the neuronal circuit and modulating social behaviors.

The present study proved that early postnatal OXT manipulation had a long-term therapeutic effect on an environmental factor-induced autistic model. Early postnatal OXT treatment has also been effective for other monogenic models of autism. Further, previous findings have shown that OXT treatment was more effective in the early stage of life than in adolescence (Penagarikano et al., 2015). In clinical studies, OXT treatment for patients with ASD is mostly concentrated during adolescence or adulthood, with a demonstrable therapeutic effect (Hollander et al., 2003; Hollander et al., 2007; Andari et al., 2010; Guastella et al., 2010; Hall et al., 2012; Rilling et al., 2014). However, the long-term efficacy of multiple or chronic OXT treatment is controversial (Tachibana et al., 2013; Guastella et al., 2015; Watanabe et al., 2015). Combining the findings regarding postnatal OXT treatment in different types of autistic models, starting the treatment at an early age might be advantageous. Further research is required for underlying mechanism that links OXT and ASD, as well as the selection of timing, duration, and route of administration for OXT treatment.

In summary, the present study provides some insights on the VPA-induced autistic model in rat, and showed that the early postnatal OXT treatment had a long-term therapeutic effects on autistic-like behaviors in these rats.

## Author Contributions

YD and HZ conceived and designed the study, performed the experiments, analyzed the data, and wrote the manuscript. RZ, JH, and SH helped to design the study and contributed to analysis with constructive discussions. RZ, JH, SH, TB, and MS reviewed and edited the manuscript and made valuable suggestions. All authors approved the final version.

## Conflict of Interest Statement

The authors declare that the research was conducted in the absence of any commercial or financial relationships that could be construed as a potential conflict of interest.
